# Reconsidering the Prognostic Role of the HALP Score in Rheumatic Mitral Stenosis

**DOI:** 10.1002/clc.70334

**Published:** 2026-04-28

**Authors:** Muhammad Talha, Sawera Gul, Muhammad Faizan Khan

**Affiliations:** ^1^ Nowshera Medical College Khyber Medical University Peshawar Pakistan; ^2^ Khyber Girls Medical College Peshawar Pakistan

To the Editor,

We read with considerable interest the recently published study by Kaya et al. [1] evaluating the prognostic value of the hemoglobin–albumin–lymphocyte–platelet (HALP) score in patients with rheumatic mitral stenosis. The authors address an important clinical need, particularly in resource‐constrained settings where simple and cost‐effective biomarkers are desirable. However, the modest discriminatory performance and the absence of independent significance in multivariable analysis suggest that interpreting HALP as a true prognostic marker do not support its interpretation as an independent prognostic marker.

First, there appears to be a conceptual overlap between a prognostic biomarker and what may instead represent a state marker. A robust prognostic biomarker is expected to provide independent and incremental predictive value beyond established clinical variables. In this study, the HALP score did not retain statistical significance after adjustment, which raises the possibility that it reflects the patient's current physiological or inflammatory condition rather than reliably predicting future risk. This distinction has been emphasized in established biomarker frameworks, where simple association is not sufficient to define prognostic utility [2]. The lack of statistical significance in multivariable analysis further undermines its role as an independent prognostic biomarker. Moreover, the modest discriminatory performance (AUC ≈ 0.64) indicates limited clinical utility.

Second, reliance on a single baseline measurement introduces a temporal limitation. The components of the HALP score are inherently dynamic and can fluctuate with changes in clinical status, treatment, and nutritional state. Models intended for long‐term risk prediction generally benefit from temporal consistency or repeated assessments. Previous work has shown that incorporating longitudinal biomarker trends can enhance predictive accuracy compared to single time‐point measurements [3, 4]. Additionally, the inclusion of intervention‐based endpoints may introduce clinician‐dependent bias, potentially confounding the assessment of true prognostic value.

Third, the biological non‐specificity of the HALP score deserves consideration. Composite indices reflecting inflammation and nutritional status are not disease‐specific and have demonstrated prognostic associations across a wide spectrum of cardiovascular conditions [5, 6, 7]. For instance, HALP has been linked with mortality in coronary heart disease, outcomes following percutaneous coronary intervention, and atrial fibrillation cohorts. Similar associations have also been reported in valvular and surgical populations. These findings suggest that HALP may primarily capture a generalized systemic vulnerability rather than mechanisms unique to rheumatic mitral stenosis, which may limit its disease‐specific predictive value.

Taken together, these observations suggest that while the HALP score is accessible and inexpensive, it more likely reflects systemic health status rather than disease‐specific risk and should not be considered a standalone prognostic tool. Future prospective studies incorporating serial measurements and evaluating incremental predictive value over established clinical and echocardiographic models would help clarify its clinical relevance. Until such evidence is available, caution may be warranted in considering HALP as a disease‐specific prognostic marker in rheumatic mitral stenosis.

## Author Contributions

All authors contributed equally to the conception and writing of this manuscript.

## Funding

The authors have nothing to report.

## Ethics Statement

The authors have nothing to report.

## Conflicts of Interest

The authors declare no conflicts of interest.

## Data Availability

Data sharing not applicable to this article as no datasets were generated or analysed during the current study.
